# Editorial: Essential Pathways and Circuits of Autism Pathogenesis

**DOI:** 10.3389/fnins.2016.00182

**Published:** 2016-04-26

**Authors:** Gül Dölen, Mustafa Sahin

**Affiliations:** ^1^Department of Neuroscience, School of Medicine, Brain Science Institute, Johns Hopkins UniversityBaltimore, MD, USA; ^2^Department of Neurology, F.M. Kirby Neurobiology Center, Harvard Medical School, Boston Children's HospitalBoston, MA, USA

**Keywords:** striatum, cerebellum, fragile X syndrome, oxytocin, vasopressin, amygdala, pathoclisis, synaptopathy

Autism spectrum disorder (ASD) is a neurodevelopmental disorder characterized by impairments in social communication skills, as well as stereotyped movements and restricted interests (DSM-5; American Psychiatric Association, 2013). Appreciation of the genetic etiology of ASD began with epidemiological studies in the 1970s, revealing the extremely high heritability of the disorder. Since then over 700 genes have been implicated in the etiology of ASD. A handful of these are highly potent rare variant mutations (e.g., Mendelian disorders like Fragile X; structural, or copy number variants, CNVs, like 16p11.2 deletion/duplication; and *de novo*, rare variant exonic mutations like chromodomain helicase DNA-binding protein 8, *CHD8*, gene mutations). Nevertheless, ASD risk mutations are also incompletely penetrant (only a subset of patients who have the mutation also have ASD), pleiotropic (all known mutations are also causes of intellectual disability, schizophrenia, and/or epilepsy), and likely highly polygenic (i.e., one characteristic is controlled by two or more genes; estimates for ASD range from 400 to 1000 genes). Accumulating evidence suggests that, in the face of this etiological complexity, we may be able to understand the emergence of key core clinical symptoms by examining a limited number of convergent biochemical pathways or brain circuits. This Frontiers Research Topics brings together a set of review articles, which explore the existing evidence supporting this view.

Although, deficits in social interactions and restrictive, repetitive patterns of behavioral output are seemingly unrelated symptom domains, growing appreciation of striatal function suggests that this brain region regulates behavioral flexibility, motivational state, goal-directed learning, and attention. The review articles by Fuccillo and Rothwell consider whether alterations in striatal physiology might be a central node mediating a range of autism-associated behaviors, including social and cognitive deficits that are hallmarks of the disorder. Similarly, the cerebellum is classically thought to control fine motor function, but more recent evidence implicates this brain region in higher cognitive functions as well. Three manuscripts critically review the hypothesis that the cerebellum is essential for many, if not most of the processes that are perturbed in ASD, including language and communication, social interactions, stereotyped behavior, motor activity and motor coordination, and higher cognitive functions (Hampson and Blatt; D'Mello and Stoodley; Mosconi et al.).

Circuit-level explanations of ASD pathogenesis are appealing because they most directly account for the emergence of clinical symptoms; however, because ASD genes are expressed across the whole brain, it is at this time unclear how specific circuits, cell types and brain regions are more likely to be involved in producing symptoms. One compelling possibility is that this pattern emerges through pathoclisis (i.e., the whole brain is exposed to the insult, in this case genetic lesion, but certain subtypes of cells, localized to distinct brain regions or circuits, are more susceptible to injury). Alternatively, the selective involvement of certain brain regions or circuits may reflect the case that these circuits are recruited as a compensatory shunting mechanism for symptoms that arise in a distributed fashion throughout multiple brain regions and circuits (this alternative is perhaps analogous to “abdominal guarding” whereby the muscles of the abdominal wall contract when there is injury to any of the organs found within the abdominal cavity). In the case of pathoclisis, future studies aimed at understanding how certain cell types become selectively susceptible may yield important pathogenic mechanisms and therapeutic targets. In the case of compensatory shunting, examining underlying, and unifying biochemical or molecular mechanisms will be critical for understanding the pathogenesis.

The first suggestion that ASD might be thought of as a “synaptopathy” was driven by the observation that the dozen or so ASD risk genes known at the time, all encode synaptic proteins, and could be linked together by biochemical signaling pathways that regulate synaptic pruning and plasticity during early post-natal development. Later, the Fragile X mental retardation protein (FMRP), which is absent in patients with Fragile X (the first identified and most common cause of ASD) binds to nearly one quarter of identified ASD candidate genes, suggesting that this protein might serve as a central node for ASD pathogenesis. Interestingly, even as we discover novel unifying mechanisms, these early speculations are also being borne out by genetic pathway analysis. Several reviews in this volume address these unifying mechanisms revealed by molecular, biochemical, and genetic pathway analysis.

Baribeau and Anagnostou summarize the known associations between the oxytocin and vasopressin neuropeptide systems and social neurocircuits in the brain. Meffert and colleagues review evidence that genetically diverse forms of ASD may be usefully parsed into entities resulting from converse patterns of growth regulation at the molecular level, which lead to the correlates of general synaptic and neural overgrowth or undergrowth (Subramanian et al.). Sell and Margolis examine the hypothesis that changes in UBE3A protein levels alter the levels of a collection of protein substrates giving rise to the unique phenotypic aspects of *UBE3A* associated ASDs. Huang and Hsueh focus *T-brain-1* (*TBR1*) and argue that this gene serves as a node for ASD pathogenesis, as well as reviews their recent evidence that *Tbr1*^+∕−^ ASD model mice show amygdalar wiring and NMDAR hypoactivity phenotypes. O'Roak and colleagues focus on genes involved in transcriptional regulation, such as chromatin modifiers and summarizes evidence that *CHD8*, a chromatin remodeling factor, may serve as a “master regulator” of a common ASD etiology (Barnard et al.). Dougherty and colleagues consider evidence from genetic pathway analysis that reveal clusters of ASD associated genes, which are involved in a handful of cellular functions, as well as the developmental time course, brain region and cell-type specificity of those functions (Kopp et al.).

We are currently at a critical juncture in ASD research. As we discover more and more pathogenic mechanisms, it is important to step back and synthesize so that we may generate novel testable hypothesis about whether and how these mechanisms may intersect to produce the common symptoms of ASD. We hope that the papers brought together in this Frontiers Research Topic will serve to stimulate that conversation and provide the readers with new ideas and perspectives toward such convergent mechanisms and circuits.

## Author contributions

All authors listed, have made substantial, direct, and intellectual contribution to the work, and approved it for publication.

### Conflict of interest statement

The authors declare that the research was conducted in the absence of any commercial or financial relationships that could be construed as a potential conflict of interest.
